# Construction and validation of a DNN-based biological age and its influencing factors in the China Kadoorie Biobank

**DOI:** 10.1007/s11357-025-01577-x

**Published:** 2025-03-07

**Authors:** Yushu Huang, Lijuan Da, Yue Dong, Zihan Li, Yuan Liu, Zilin Li, Xifeng Wu, Wenyuan Li

**Affiliations:** 1 Center of Clinical Big Data and Analytics of the Second Affiliated Hospital, and Department of Big Data in Health Science School of Public Health, Zhejiang University School of Medicine, Hangzhou, Zhejiang China; 2 School of Mathematics and Statistics and KLAS, Northeast Normal University, Changchun, Jilin China; 3 Zhejiang Provincial Key Laboratory of Intelligent Preventive Medicine, Hangzhou, Zhejiang China

**Keywords:** Deep learning, Biological age, Age acceleration, Influencing factors, Morbidity, Mortality

## Abstract

Biological age is an important measure of aging that reflects an individual’s physical health and is linked to various diseases. Current prediction models are still limited in precision, and the risk factors for accelerated aging remain underexplored. Therefore, we aimed to develop a precise biological age and assess the impact of socio-demographic and behavioral patterns on the aging process.We utilized Deep Neural Networks (DNN) to construct biological age from participants with physical examinations, blood samples, and questionnaires data from the China Kadoorie Biobank (CKB) between June 2004 and December 2016. △age, calculated as the residuals between biological age and chronological age, was used to investigate the associations of age acceleration with diseases. Socio-demographics (gender, education attainment, marital status, household income) and lifestyle characteristics (body mass index [BMI], smoking, drinking, physical activity, and sleep) were also assessed to explore their impact on age acceleration. 18,261 participants aged 57 ± 10 years were included in this study. The DNN-based biological age model has demonstrated accurate predictive performance, achieving a mean absolute error of 3.655 years. △age was associated with increased risks of various morbidity and mortality, with the highest associations found for circulatory and respiratory diseases, with hazard ratios of 1.033 (95% CI: 1.023, 1.042) and 1.078 (95% CI: 1.027, 1.130), respectively. Socio-demographics, including being female, lower education, widowed or divorced, and low household income, along with behavioral patterns, such as being underweight, insufficient physical activity, and poor sleep, were associated with accelerated aging. Our DNN model is capable of constructing a precise biological age using commonly collected data. Socio-demographics and lifestyle factors were associated with accelerated aging, highlighting that addressing modifiable risk factors can effectively slow age acceleration and reduce disease risk, providing valuable insights for interventions to promote healthy aging.

**Supplementary Information:**

The online version contains supplementary material available at 10.1007/s11357-025-01577-x.

## Introduction

Aging is one of the primary risk factors for various age-related diseases and can be assessed by biological age. Individuals may have different biological ages even if their chronological ages are the same, indicating variability in their rates of aging and differing susceptibility to death and diseases [1]. Studies have shown that age acceleration is linked to greater physiological function deterioration across multiple systems and organs, resulting in higher risks of multimorbidity and mortality, including cancer, heart disease, cerebrovascular disease, respiratory disease, diabetes, nephrosis, and depression [2–5]. Taken together, the strong association between age acceleration and disease suggests that biological age is a better indicator of an individual’s health condition.

In recent years, various indicators for measuring biological age have been developed, including those based on omics data, physiological markers, and functional measures [6–8]. However, these methods still have limitations in clinical applications due to challenges such as data inaccessibility, high costs, or technical complexity. Therefore, using commonly collected data to calculate a more accurate biological age presents significant research value. For instance, Qiu et al. [9] employed Cox proportional hazard gradient boosted trees with blood tests and questionnaire data from the UK Biobank to construct a biological age, which showed a strong correlation with chronological age, with a correlation coefficient of 0.7867. Bernard et al. [10] developed a machine learning-based biological age using blood samples from the National Health and Nutrition Examination Survey (NHANES) study, achieving a mean absolute error (MAE) of 8.1 years. Nevertheless, these methods have limitations due to insufficient accuracy. Therefore, applying deep learning models for assessing biological age is a promising solution, as it offers significant advantages in handling large-scale datasets, automatically extracting features, and enhancing computational efficiency [7, 11].

Moreover, evidence from recent studies indicates that age acceleration is closely associated with adverse socio-demographic and behavioral factors [3, 12–15], thereby affecting health in later years. For instance, a study conducted in Italy suggested that lifestyle characteristics (e.g., body mass index (BMI), smoking, alcohol consumption, and physical activity) and socioeconomic position (e.g., education level, employment class, household income, housing status) together account for approximately 48% of the total variance in accelerated aging [11]. Nevertheless, most relevant researches were conducted in Europe and the United States [12, 16], with few studies carried out in Asia, especially in China [7, 17]. Exploring the relationship between age acceleration, its influencing factors, and health outcomes is necessary in China, which is experiencing unprecedented aging that results in significant social, economic, and healthcare burdens.

To address these knowledge gaps, the present study was conducted using questionnaire, physical examination, and laboratory tests data from the China Kadoorie Biobank (CKB), a prospective cohort of Chinese adults. This study aimed to construct a precise biological age and examine the association of age acceleration with morbidity and mortality, as well as the influencing factors, which have not been fully investigated in previous studies.

## Methods

### Study participants

This was a prospective cohort study using the CKB data. The baseline survey of CKB was conducted between 2004 and 2008, recruiting over 0.5 million participants aged 30 to 79 from the general population across five cities and five rural areas in China. The program was designed to assess the lifestyle, environmental, and genetic risk factors, by combining questionnaires, physiological measurements, and blood and biological samples, with the goal of identifying key determinants of health outcomes. A total of 18,261 participants with available biomarkers were eligible for this study. The CKB study was approved by the Ethical Review Committee of the Chinese Center for Disease Control and Prevention (Beijing, China) and the Oxford Tropical Research Ethics Committee at the University of Oxford (Oxford, UK). The current analysis was approved by the Ethical Review Committee of School of Public Health, Zhejiang University School of Medicine. Written informed consent was provided by all participants. Details have been presented previously [18, 19].

### Morbidity and mortality

The disease status was self-reported by participants, and the death records were collected from the local Disease Surveillance Point death registries [19]. The cause of death was primarily obtained from official death certificates and medical records [20]. In this study, the mortality data included final vital status, underlying leading cause of death, and follow-up time. For survivors, the follow-up time extended until the end of the mortality follow-up period (December 31, 2016), while for deceased individuals, it was calculated from the date of interview to the date of death or loss to follow-up. The causes of death were classified using the 10th International Classification of Diseases (ICD-10): malignant neoplasms (C00-C97), endocrine, nutritional and metabolic diseases (E00-E90), including diabetes mellitus (E10-E14), mental and behavioral disorders (F00-F99), disease of the circulatory system (I00-I99), including hypertension (I10-I15), ischemic heart diseases (I20-I25), and cerebrovascular diseases (I60-I69), disease of the respiratory system (J00-J99), including chronic lower respiratory diseases (J40-J47), and disease of the genitourinary system (N00-N99).

### Biological age construction and validation

Based on previous literatures and the availability of samples, we selected 18 biochemical, 7 physical and 26 questionnaire indicators to construct a Deep Neural Network (DNN) model for the prediction of biological age (Table. S1). The result of Pearson rank correlation coefficients is shown in Fig. S1. The model architecture consists of 5 hidden layers with 256, 128, 64, 10, and 1 neuron, respectively. Each layer employs the ReLU activation function, followed by a Dropout rate of 30% for regularization in the first four layers. The final output layer consists of a single neuron for continuous age prediction. The network was trained using the Adam optimizer, with a learning rate of 0.01, a batch size of 1024, and Mean Squared Error (MSE) as the loss function. Training was performed for 1000 epochs, using an 80% random extraction of the dataset (N = 14,609), with a 20% internal validation set to mitigate overfitting. To enhance model robustness, we conducted 10 independent test runs, averaging the results for evaluation. The model’s performance was assessed on the test set (remaining 20% of the dataset, N = 3,652) using Pearson correlation coefficients and MAE between chronological age and biological age as evaluation metrics.

In addition, to quantify the aging rate of different participants, we regressed their biological age on chronological age and calculated the residual value, which was defined as “△age”. If △age is greater than 0, it indicates accelerated aging for the individual (assign to the “accl” group), if △age is less than 0, it indicates non-accelerating aging (assign to the “normal” group).

### Influencing factors of biological age

As described in previous studies [17, 21], five influencing factors were primarily considered, including BMI, smoking, drinking, physical activity, and sleep.

BMI was calculated by dividing a person’s weight in kilograms by the square of their height in meters, and was classified into four groups, with BMI < 18.5 kg/m^2^ classified as underweight, 18.5–25.0 kg/m^2^ as normal, 25.0–30.0 kg/m^2^ as overweight, ≥ 30 kg/m^2^ as obesity.

Smoking status was classified into three categories, including never, former, and current. For drinking, we calculated the weekly pure alcohol intake based on the type and frequency of alcohol consumption (measured in g/week). The average alcohol content (v/v) for different beverages was as follows: beer (4%), rice wine (10%), wine (12%), spirits with ≤ 50% alcohol (40%), and spirits with ≥ 50% alcohol (60%). We classified alcohol consumption into four categories: never drinking, former drinking (more than 12 months ago), light drinking (< 210 g of pure alcohol per week for males or < 105 g per week for females), and heavy drinking (≥ 210 g of pure alcohol per week for males or ≥ 105 g per week for females) [17].

To explore the effect of physical activity, individuals were divided into three groups (low, moderate, high) based on metabolic equivalent of task (MET) levels, using tertiles and stratified by gender [22]. The MET ranges were < 9.9 MET-hours/day, 9.9–23.0 MET-hours/day, and > 23.0 MET-hours/day for males, and < 10.7 MET-hours/day, 10.7–19.2 MET-hours/day, and > 19.2 MET-hours/day for females.

Sleep quality was assessed by constructing a sleep score based on several sleep-related indicators, including sleep duration, delayed or fitful sleep, sleep affecting daily life, sleep needing medicine, waking up too early, and daytime naps [23]. First, the reference values were set as follows: “7 ~ 8 hours” for sleep duration, “No” for delayed or fitful sleep, sleep affecting daily life, sleep needing medicine, waking up too early, and daytime naps. Then, these indicators, along with chronological age, were incorporated into a Cox model with mortality as the outcome to obtain the coefficients for each sleep indicator. The standardized coefficients were then rounded to the nearest integer, and the final sleep score was calculated by summing these values. Detailed scoring criteria are provided in Table. S2.

### Covariates

Information on participants’ socio-demographic characteristics included age, gender, education attainment, marital status, and household income. Education attainment was categorized into primary school or lower, middle school, and high school or higher. Marital status included with spouse, widowed/divorced, and never married. Household income was divided into four categories, namely less than 10,000 Chinese yuan (CNY)/year, 10,000–19,999 CNY/year, 20,000–34,999 CNY/year, and 35,000 CNY/year or more.

### Statistical analysis

We presented the baseline characteristics of the participants as mean (standard deviation [SD]) for continuous variables and as count (percentage) for categorical variables. Multiple imputations using random forests were applied to impute missing values. Receiver Operating Characteristic (ROC) curves based on multivariable Cox regressions were used to compare the predictive power of chronological age and biological age for all-cause mortality.

We employed multivariable Cox regressions, which satisfied the proportional hazards assumption, to assess the risk for both all-cause and cause-specific mortality, and reported the hazard ratio (HR) for each additional year of △age. Our analysis included three models, the initial model (model 1) was unadjusted, model 2 was adjusted for age and gender, and model 3 was adjusted for age, gender, education attainment, marital status, household income, BMI, smoking, drinking, physical activity, and sleep. Additionally, we explored the relationship between △age and morbidity (diabetes, hypertension, rheumatic heart disease) at baseline using multiple logistic regression, adjusting for the same covariates. We calculated the risk of disease associated with each additional year of △age and reported the odds ratio (OR). Furthermore, a mutually adjusted multiple linear regression was conducted to analyze the associations between behavioral factors, covariates and △age.

The DNN model was built using Pytorch (version 2.1.1) in Python (version 3.11.5). Data cleaning and analysis were carried out using R software (version 4.3.2). The Cox model was applied using the “survival” package. A two-tailed P-value less than 0.05 was considered statistically significant.

## Results

### Baseline characteristics of participants

The baseline characteristics of the participants are shown in Table 1. Of the 18,261 participants, 12,810 (70.2%) were classified as healthy individuals (without any clarified diagnosed disease), 5,451 (29.8%) were classified as unhealthy individuals. In the whole population, 5,621 (30.8%) deaths occurred during a median follow-up period of 8.9 ± 3.1 years. In the comparison between the healthy and unhealthy groups, the average age of the unhealthy group (59 ± 9.2 years) was higher than that of the healthy group (56 ± 11.0 years). Additionally, the unhealthy group has a higher proportion of males, individuals with lower education levels, those widowed or divorced, and higher household income.

**Table 1:** Baseline characteristics of study participants

	All (N = 18,261)	Healthy (N = 12,810)	Unhealthy (N = 5,451)
Death, N (%)			
Yes	5,621 (30.8%)	3,381 (26.4%)	2,240 (41.1%)
No	12,640 (69.2%)	9,429 (73.6%)	3,211 (58.9%)
Follow-up time, mean (SD), years	8.9 (3.1)	9.1 (2.9)	8.3 (3.3)
Age, mean (SD), years	57 (10.0)	56 (11.0)	59 (9.2)
Gender			
Male	8,986 (49.2%)	6,115 (47.7%)	2,871 (52.7%)
Female	9,275 (50.8%)	6,695 (52.3%)	2,580 (47.3%)
Education attainment			
Primary school or lower	11,262 (61.7%)	7,790 (60.8%)	3,472 (63.7%)
Middle school	4,216 (23.1%)	3,040 (23.7%)	1,176 (21.6%)
High school or higher	2,783 (15.2%)	1,980 (15.5%)	803 (14.7%)
Marital status			
With spouse	15,785 (86.4%)	11,137 (86.9%)	4,648 (85.3%)
Widowed/divorced	2,293 (12.6%)	1,531 (12.0%)	762 (14.0%)
Never married	183 (1.0%)	142 (1.1%)	41 (0.8%)
Household income, CNY/year			
< 10,000	7,094 (38.8%)	5,105 (39.9%)	1,989 (36.5%)
10,000–19,999	5,559 (30.4%)	3,917 (30.6%)	1,642 (30.1%)
20,000–34,999	3,579 (19.6%)	2,436 (19.0%)	1,143 (21.0%)
≥ 35,000	2,029 (11.1%)	1,352 (10.6%)	677 (12.4%)
BMI, kg/m^2^			
Underweight	1,023 (5.6%)	855 (6.7%)	168 (3.1%)
Normal	11,220 (61.4%)	8,463 (66.1%)	2,757 (50.6%)
Overweight	5,233 (28.7%)	3,128 (24.4%)	2,105 (38.6%)
Obesity	785 (4.3%)	364 (2.8%)	421 (7.7%)
Smoking			
Never	9,587 (52.5%)	6,547 (51.1%)	3,040 (55.8%)
Former	1,416 (7.8%)	880 (6.9%)	536 (9.8%)
Current	7,258 (39.7%)	5,383 (42.0%)	1,875 (34.4%)
Drinking			
Never	8,172 (44.8%)	5,456 (42.6%)	2,716 (49.8%)
Former	528 (2.9%)	268 (2.1%)	260 (4.8%)
Light	7,845 (43.0%)	5,765 (45.0%)	2,080 (38.2%)
Heavy	1716 (9.4%)	1,321 (10.3%)	395 (7.2%)
Physical activity			
Low	6,088 (33.3%)	3,871 (30.2%)	2,217 (40.7%)
Moderate	6,087 (33.3%)	4,267 (33.3%)	1,820 (33.4%)
High	6,086 (33.3%)	4,672 (36.5%)	1,414 (25.9%)
Sleep score	3.1 (2.3)	3.0 (2.3)	3.2 (2.4)

The healthy group was defined as those who did not have any clarified diagnosed disease at baseline. SD: standard deviation, BMI: body mass index, CNY: Chinese yuan. Continuous variables were presented as the mean (SD), and categorical variables were presented as n (%)

### DNN model performance

**Fig. 1:**
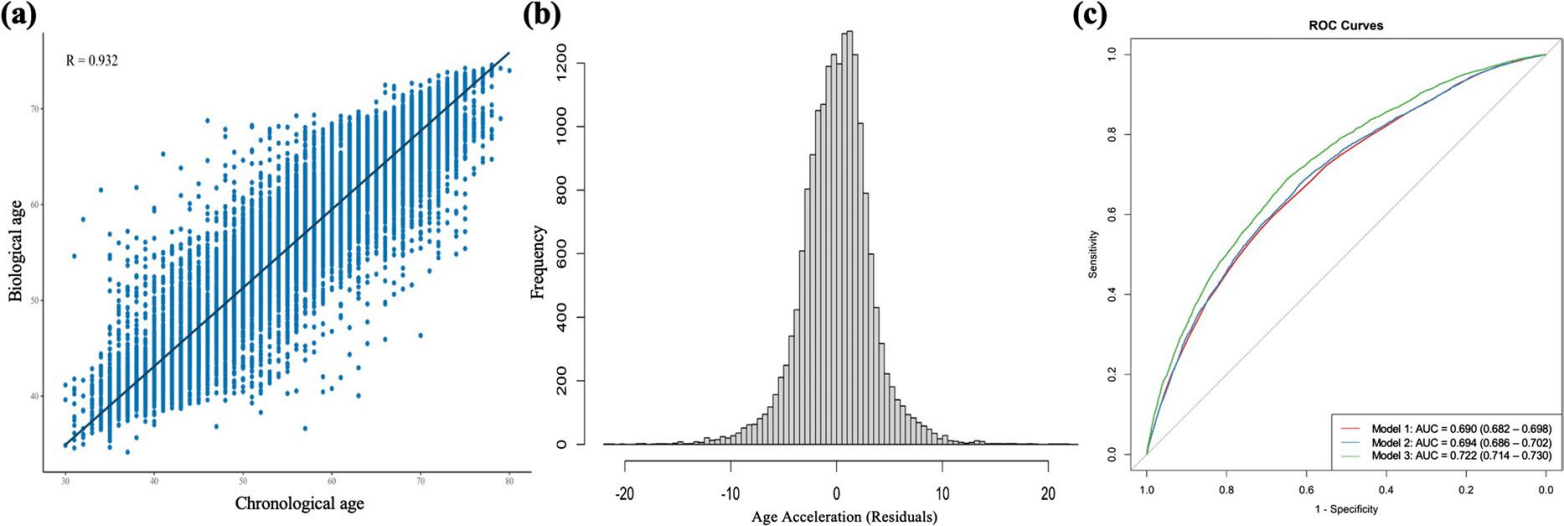
DNN model performance. **a** The relationship between biological age and chronological age. Biological age is highly correlated with chronological age, with a correlation coefficient of 0.932. **b** Distribution of age acceleration. The mean of age acceleration is 0.00 years, with a standard deviation of 4.40 years. **c** Receiver operating characteristic (ROC) curves and the area under the curve (AUC) for all-cause mortality prediction. Model 1 was built based on chronological age, model 2 was based on biological age, model 3 was based on biological age, gender, education attainment, marital status, household income, body mass index (BMI), smoking, drinking, physical activity, and sleep

Biological age is highly correlated with chronological age, with a correlation coefficient of 0.932, and the mean of age acceleration is 0.00 years, with a standard deviation of 4.40 years, as shown in Fig. 1 (a) and (b). The best-performing DNN model accurately predicts biological age with an MAE of 3.655 years on the test set. In predicting all-cause mortality, the area under the curve (AUC) for chronological age was 0.690 (95% confidence interval [CI]: 0.682, 0.698) and that for biological age was 0.694 (95% CI:0.686, 0.702). After adjusting for gender, education level, marital status, BMI, smoking, drinking, physical activity, and sleep, the AUC value only slightly increased, suggesting that biological age is an effective predictor of death (Fig. 1 (c)). Additionally, we found biological age was a stronger predictor of mortality than chronological age. For each one-year increase in biological age, the risk of death increases by 5.2% (95% CI: 4.8%, 5.6%), while for chronological age, the risk of death increases by 4.4% (95% CI: 4.0%, 4.7%) (Table. S3).

### Associations of age acceleration with mortality

**Fig. 2:**
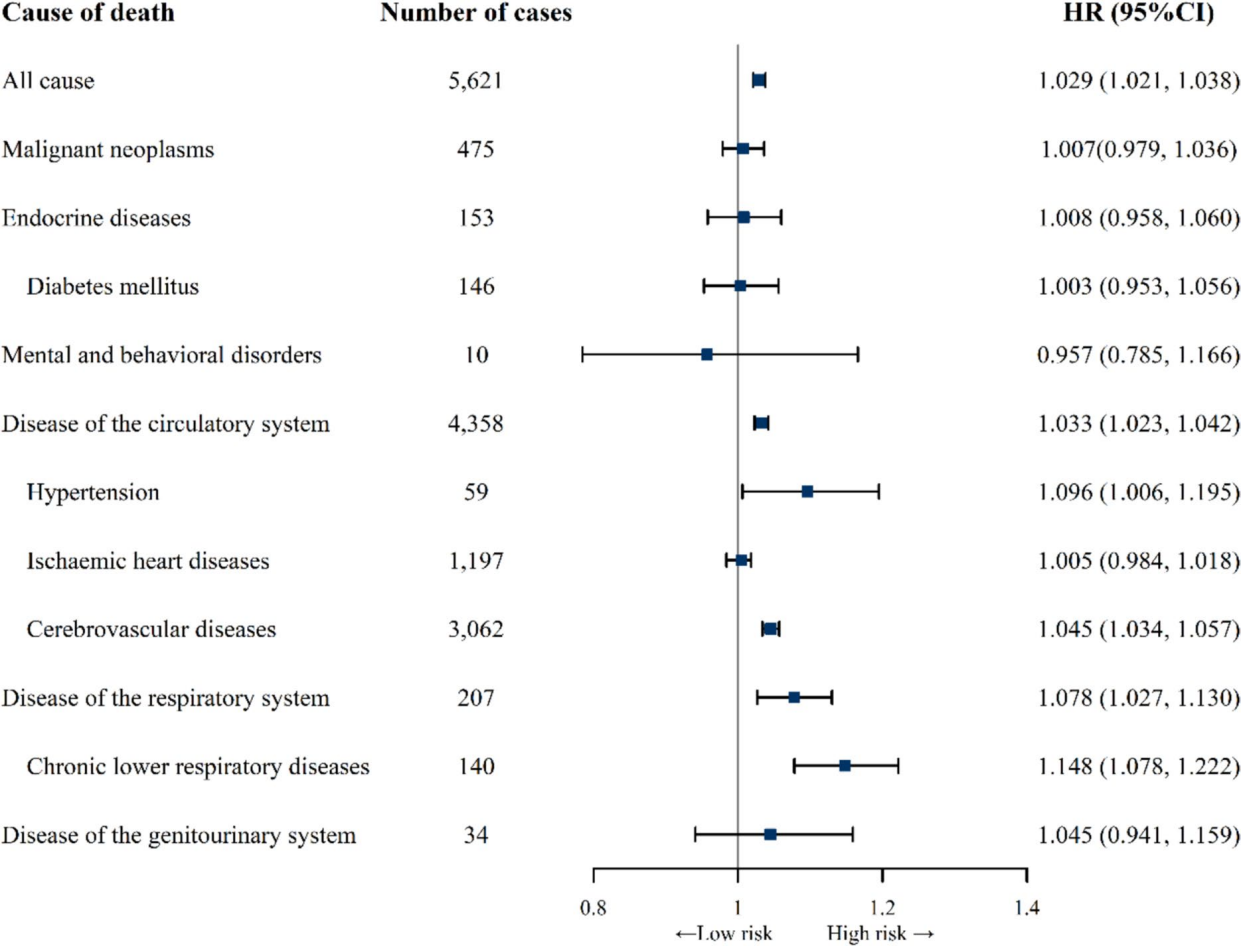
Association of each 1-year increment of age acceleration with cause-specific mortality. HR: hazard ratio, CI: confidence interval. Cox models were adjusted for chronological age, gender, education attainment, marital status, household income, body mass index (BMI), smoking, drinking, physical activity, and sleep

In the overall population, age acceleration was associated with an increased risk of all-cause mortality, with the adjusted HR for each one-year increment of △age being 1.029 (95% CI: 1.021, 1.038), as depicted in Fig. 2. Among all diseases, associations with △age were statistically significant for circulatory diseases and respiratory diseases. △age (per one-year increment) was associated with a 3.3% (95% CI: 2.3%, 4.2%) increase in the risk of circulatory mortality and a 7.8% (95% CI: 2.7%, 13.0%) increase in the risk of respiratory mortality. Notably, the highest mortality risk was observed in hypertension (HR: 1.096, 95% CI: 1.006, 1.195), cerebrovascular diseases (HR: 1.045, 95% CI: 1.034, 1.057), and chronic lower respiratory diseases (HR: 1.148, 95% CI: 1.078, 1.222). However, associations for malignant neoplasms, endocrine diseases, mental and behavioral disorders, as well as genitourinary diseases, were not statistically significant.

### Associations of age acceleration with morbidity

As Table 2 showed, △age was associated with both diabetes and hypertension. These associations remained stable after adjusting for age, gender, education attainment, marital status, household income, BMI, smoking, drinking, physical activity, and sleep. For each additional year of △age, the risk of diabetes increased by 2.6% (95% CI: 0.9%, 4.3%), and the risk of hypertension increased by 1.9% (95% CI: 0.8%, 3.0%). However, the association with rheumatic heart disease was not significant (OR: 1.063, 95% CI: 0.980, 1.147).

**Table 2:** Association of each 1-year increment of age acceleration with morbidity

Model	Diabetes	Hypertension	Rheumatic heart disease
1	1.020 (1.004, 1.035)	1.010, (1.000, 1.020)	1.062 (0.986, 1.139)
2	1.022 (1.006, 1.038)	1.012 (1.001, 1.022)	1.076 (0.996, 1.159)
3	1.026 (1.009, 1.043)	1.019 (1.008, 1.030)	1.063 (0.980, 1.147)

Model 1 was unadjusted, model 2 was adjusted for age and gender, model 3 was adjusted for age, gender, education attainment, marital status, household income, body mass index (BMI), smoking, drinking, physical activity, and sleep

### Associations of influencing factors with age acceleration

According to the results of multiple linear regression mutually adjusted for covariates, accelerated aging is positively associated with multiple socio-demographic factors and behavioral patterns. For instance, compared to those with a normal BMI, underweight individuals showed a significant increase in age acceleration by 0.485 (95% CI: 0.275, 0.694) years, whereas being overweight and obese resulted in decreases in △age by −0.411 (95% CI: −0.520, −0.302) years and −0.646 (95% CI: −0.884, −0.408) years, respectively. Compared to never drinkers, light alcohol consumption was associated with a decrease in △age by −0.280 (95% CI: −0.391, −0.169) years, while former drinking and heavy alcohol consumption showed no statistically significant effect. Regarding physical activity, compared to moderate levels, low activity was found to potentially accelerate aging, while high activity was linked to decelerated aging, with β values of 0.559 (95% CI: 0.441, 0.677) and −0.849 (95% CI: −0.969, −0.73), respectively. For each one-point increase in sleep score, there was a corresponding increase in △age of 0.068 (95% CI: 0.047, 0.089) years. However, there was no statistically significant association between smoking status and age acceleration. Additionally, the association between adverse socio-demographic factors and age acceleration were stronger in males, individuals with lower education attainment, those who were widowed or divorced, and participants with low household income, as detailed in Table 3 and Table S4.

**Table 3:** Associations of socio-demographic and lifestyle factors with age acceleration

Variable	β (95% CI)	P value
Gender		
Male	reference	
Female	−0.641 (−0.805, −0.477)	< 0.001
Education attainment		
Primary school or lower	reference	
Middle school	−0.494 (−0.620, −0.368)	< 0.001
High school or higher	−1.126 (−1.277, −0.975)	< 0.001
Marital status		
With spouse	reference	
Widowed/divorced	0.687 (0.536, 0.837)	< 0.001
Never married	−0.651 (−1.131, −0.172)	< 0.05
Household income		
< 10,000	reference	
10,000–19,999	−0.278 (−0.395, −0.160)	< 0.001
20,000–34,999	−0.346 (−0.482, −0.210)	< 0.001
≥ 35,000	−0.250 (−0.418, −0.083)	< 0.001
BMI		
Underweight	0.485 (0.275, 0.694)	< 0.001
Normal	reference	
Overweight	−0.411 (−0.520, −0.302)	< 0.001
Obesity	−0.646 (−0.884, −0.408)	< 0.001
Smoking		
Never	reference	
Former	−0.007 (−0.230, 0.216)	0.954
Current	−0.109 (−0.272, 0.055)	0.194
Drinking		
Never	reference	
Former	0.119 (−0.177, 0.414)	0.432
Light	−0.280 (−0.391, −0.169)	< 0.001
Heavy	−0.179 (−0.365, 0.006)	0.058
Physical activity		
Low	0.559 (0.441, 0.677)	< 0.001
Moderate	reference	
High	−0.849 (−0.969, −0.730)	< 0.001
Sleep score	0.068 (0.047, 0.089)	< 0.001

CI: confidence interval, BMI: body mass index. Multiple linear regression models were used to impute the β (95% CI). When evaluating each factor, the others were also adjusted in the regression models

## Discussion

In this large-scale prospective cohort study, we developed and validated a highly precise biological age model using DNN with routinely collected multidimensional data, and demonstrated strong and consistent associations of this novel biological age biomarker with morbidity and mortality risk. Additionally, we identified multiple socio-demographic and lifestyle factors contributing to accelerated aging. To our knowledge, this is among the first large-scale studies to apply deep learning method in assessing biological aging in a nationwide cohort in China. Our findings not only provide a novel tool for biological age estimation, but also shed light on potential intervention targets to mitigate age-related health risks, and to improve overall healthy longevity.

While previous studies have primarily used physical measurements and biochemical indicators to calculate biological age [17], we enhanced this approach by incorporating questionnaire data, including information on diet, sleep, and mental state. Based on this composite dataset, we achieved the smallest reported MAE of 3.655 years, indicating that combining questionnaire data significantly improves predictive accuracy. Moreover, we replaced the commonly used Klemera-Doubal method (KDM) with DNN, since DNN can integrate individuals’ disease or mortality status and offer better stability [24, 25]. Our results are supported by previous studies, such as the work by Putin et al. [25], who found that DNN outperforms other machine learning algorithms in predicting biological age using anonymized blood biochemistry records, achieving a coefficient of determination of 0.8 and a MAE of 6.07 years. And Hung et al. [26] found that DNN can achieve a 92% AUC in stroke prediction and requires less training data, demonstrating better predictive performance and stability than other machine learning. Therefore, utilizing DNN with strong collective performance to analyze multidimensional data and reveal complex relationships between biomarkers has the potential to provide more accurate aging predictions.

Consistent with previous research, we found that accelerated aging increases the risk of mortality, particularly for circulatory and respiratory systems, including hypertension, cerebrovascular diseases, and chronic lower respiratory diseases [27–29]. In the context of age acceleration, detrimental changes in cardiac structure, such as reduced left ventricular mass, decreased overall ventricular volume, and diminished stroke volumes across ventricles and atria, are significantly associated with circulatory system diseases [30]. And decreases in lung function, including lung capacity and elasticity, airway patency, and expiratory muscle strength, are associated with respiratory system diseases [31]. Additionally, the occurrence of chronic diseases, such as diabetes, is also associated with accelerated aging. As previous research has suggested, individuals with diabetes exhibit signs of “accelerated” aging approximately 10 years before diagnosis [32]. These findings indicate that signs of accelerated aging could serve as early biomarkers for chronic diseases and mortality, aiding in early intervention and management. However, we did not observe an association between age acceleration and cancer, which aligns with the finding of Gialluisi [33]. Although previous findings have linked DNA methylation age to an increased risk of colorectal [34] and breast cancer [35], it is challenging to determine whether this is due to the limited analysis of diverse cancer cases, which inherently impacts statistical power. Additionally, more research is needed to determine the association between age acceleration and endocrine diseases [36], mental and behavioral disorders [3], and genitourinary diseases [37], as there is currently no unified consensus on these relationships.

Of note, we observed that adverse factors such as underweight, insufficient physical activity, and poor sleep were associated with age acceleration. Our results indicated that aging becomes more pronounced with a decrease in BMI, which may be attributed to nutritional deficiencies and reduced basal metabolic rates, leading to a decline in the function of various bodily systems and thus accelerating the aging process. However, we also found that overweight or obesity may slow aging, which is inconsistent with other studies [38, 39]. This discrepancy may be due to the presence of metabolic reserves in individuals with higher BMI, which could provide additional energy during periods of illness, potentially mitigating the effects of aging. Further studies involving more diverse populations are needed to confirm the role of BMI in age acceleration. Additionally, we found that insufficient physical activity increased disease risk, aligning with previous research [7]. This effect may be due to reduced immune system function and impaired autophagy affecting mitochondrial function [40]. Poor sleep was associated with increased risks of many age-related outcomes, including stroke, hypertension, and diabetes [41, 42]. For instance, other studies suggested that maintaining 7–9 hours of sleep was related to a delay in biological aging [7]. Additionally, our study found that moderate alcohol consumption may help slow aging, which is consistent with findings from a multi-ethnic study [43]. However, we did not observe a significant association between smoking and accelerated aging. In contrast, another study conducted in China found a positive correlation [7], suggesting the need for further research to clarify the role of smoking in the aging process. Furthermore, our findings highlight the critical importance of addressing structural inequalities, such as improving public education and increasing household incomes, which are fundamental causes of health disparities and can help enhance overall health.

### Strengths and limitations

The present study has some strengths. We used a large cohort covering wide geographic areas throughout China, where the large sample size and long follow-up period ensured reliable results and facilitated the observation of the associations between biological age acceleration and various aging-related outcomes from both cross-sectional and longitudinal perspectives. We developed a composite biological age indicator by integrating biochemical, physical, and notably questionnaire data, which enhanced predictive accuracy and built upon previous findings on biomarkers of aging. Additionally, we simultaneously explored the relationship between socio-demographic and behavioral patterns and age acceleration, offering valuable insights for designing interventions aimed at mitigating the effects of aging.

Our study has several limitations. First, we did not collect data on the incidence date of chronic diseases, therefore we were unable to validate the causal relationship between age acceleration and behavioral factors, nor can we determine how these factors influence individual aging trajectories. Second, due to the constraints in data availability, the predictors that could be used to estimate biological age were limited to routinely collected data. Nevertheless, we constructed an accurate biological age using a DNN model, achieving a low MAE of 3.655 years. Third, our study was conducted in the Chinese population, the exact modeling parameters may not be generalizable to populations with different ethnic or socio-demographic characteristics. However, we proved the eligibility of this biological age estimation framework and can be applied to other populations.

## Conclusions

In summary, this study developed an accurate biological age using DNN with multidimensional data, which proved to be a reliable predictor of disease and death risk. Accelerated aging was significantly associated with morbidity and mortality across various diseases, particularly in the circulatory and respiratory systems. Additionally, we identified several socio-demographic and behavioral patterns that were strongly associated with accelerated aging, suggesting potential targeted health-promoting measures that may help reduce age-related disease burden.

## Supplementary Information

Below is the link to the electronic supplementary material.

Supplementary file1 (DOCX 0.99 MB)

## Data Availability

The database and algorithm source codes used in this study are available upon reasonable request by contacting the corresponding author.
