# Supplementary material for: Construction and validation of a DNN-based biological age and its influencing factors in the China Kadoorie Biobank
Source: GeroScience. 2025 Mar 7;47(3):4241–52. doi: 10.1007/s11357-025-01577-x (PMC12181170; doi:10.1007/s11357-025-01577-x)
Supplement: Supplementary file 1 — Supplementary file1 (DOCX 0.99 MB) [file 11357_2025_1577_MOESM1_ESM.docx]

**Fig. S1 Pearson rank correlation coefficients.**

**Table. S1 Indicators used for DNN model construction.**

| Category | Variable | Unit | Description |
| --- | --- | --- | --- |
| Biochemical | Albumin | Grams/Liter | Albumin value |
|  | Alanine Aminotransferase | Units/Liter | Alanine Aminotransferase value |
|  | Gamma Glutamyl Transferase | Units/Liter | Gamma Glutamyl Transferase value |
|  | Creatinine | Micromoles/Liter | Creatinine value |
|  | Cystatin C | Milligrams/Liter | Cystatin C value |
|  | Uric Acid | Micromoles/Liter | Uric Acid value |
|  | Triglycerides | Millimoles/Liter | Triglycerides value |
|  | Apolipoprotein A1 | Milligrams/Deciliter | Apolipoprotein A1 value |
|  | Apolipoprotein B | Milligrams/Deciliter | Apolipoprotein B value |
|  | Lipoprotein a | Nanomoles/Liter | Lipoprotein (a) value |
|  | Cholesterol | Millimoles/Liter | Cholesterol value |
|  | High sensitivity C reactive protein | Milligrams/Liter | High sensitivity C-reactive protein value |
|  | Random blood sugar | mmol/Liter (×10) | Random blood sugar result from blood test |
|  | Aspartate Aminotransferase | Units/Liter | Aspartate Aminotransferase value |
|  | Fibrinogen | Grams/Liter | Fibrinogen value |
|  | 25-hydroxyvitamin D | Nanograms/Milliliter | 25-hydroxyvitamin D value |
|  | High Density Lipoprotein | Millimoles/Liter | High-Density Lipoprotein value |
|  | Low Density Lipoprotein | Millimoles/Liter | Low-Density Lipoprotein value |
| Physical | Body Mass Index | kg/m^2^ | Body Mass Index, Calculated from measured height and weight |
|  | Waist hip ratio | mm/mm | Waist:hip ratio |
|  | Fat% | Percentage (×10) | Fat% |
|  | Diastolic blood pressure | mmHg | Mean Diastolic blood pressure measurement (of the two taken) |
|  | Systolic blood pressure | mmHg | Mean Systolic blood pressure measurement (of the two taken) |
|  | Heart rate | bpm | Mean heart rate measurement (of the two taken) |
|  | Forced Expiratory Volume in 1 second | liters (×100) | Max Forced Expiratory Volume in 1 second measurement (of the two taken) |
| Questionnaire | Gender | Categorical | Gender |
|  | Education | Categorical | What is the highest level of school education you ever received? |
|  | Marital status | Categorical | What is your current marital status? |
|  | Income | yuan/year | What is the total income last year in your household? |
|  | Drinking | Categorical | Drinking status |
|  | Smoking | Categorical | Smoking status |
|  | Metabolic Equivalent of Task | MET-hours/day | Total daily physical activity (Metabolic Equivalent of Task) |
|  | Rice dietary frequency | Categorical | During the past 12 months, about how often did you eat rice? |
|  | Wheat dietary frequency | Categorical | During the past 12 months, about how often did you eat wheat? |
|  | Other staple dietary frequency | Categorical | During the past 12 months, about how often did you eat other staple foods (corn, millet etc.)? |
|  | Meat dietary frequency | Categorical | During the past 12 months, about how often did you eat meat? |
|  | Poultry dietary frequency | Categorical | During the past 12 months, about how often did you eat poultry? |
|  | Fish dietary frequency | Categorical | During the past 12 months, about how often did you eat fish/sea food? |
|  | Eggs dietary frequency | Categorical | During the past 12 months, about how often did you eat fresh eggs? |
|  | Fresh vegetables dietary frequency | Categorical | During the past 12 months, about how often did you eat fresh vegetables? |
|  | Soybean dietary frequency | Categorical | During the past 12 months, about how often did you eat soybean products? |
|  | Preserved vegetables dietary frequency | Categorical | During the past 12 months, about how often did you eat preserved vegetables? |
|  | Fruit dietary frequency | Categorical | During the past 12 months, about how often did you eat fresh fruit? |
|  | Dairy dietary frequency | Categorical | During the past 12 months, about how often did you eat dairy products (milk, yogurt)? |
|  | Sleep affecting daily life | Categorical | During the past month, did you have difficulty staying alert while at work, eating or meeting people during daytime for 3 or more days each week? |
|  | Daytime naps | Categorical | Do you usually take a daytime nap? |
|  | Delayed or fitful sleep | Categorical | During the past month, did you take >30 minutes to fall asleep after going to bed or wake up in the middle of the night for 3 or more days each week? |
|  | Sleep duration | hours | How many hours do you typically sleep per day (including naps)? |
|  | Sleep needing medicine | Categorical | During the past month, did you need to take medicine (including herbal or sleeping pills) at least once a week to help sleep? |
|  | Snoring | Categorical | Do you snore during sleep? |
|  | Waking up too early | Categorical | During the past month, did you wake up early and not be able to go back to sleep for 3 or more days each week? |
|  | CIDI score | 0 to 7 (where 7 is most severe) | Based their CIDI A questionnaire answers, how severe is this participant’s depression? |

**Table. S2 Scoring Criteria for sleep quality assessment**

| Variable | Variable type | Value | Score |
| --- | --- | --- | --- |
| Sleep duration | Categorical | >=9 hours | 2 |
|  |  | 7~8 hours (ref) | 0 |
|  |  | 5~6 hours | 3 |
|  |  | <5 hours | 7 |
| Delayed or fitful sleep | Categorical | No (ref) | 0 |
|  |  | Yes | 1 |
| Sleep affecting daily life | Categorical | No (ref) | 0 |
|  |  | Yes | 1 |
| Sleep needing medicine | Categorical | No (ref) | 0 |
|  |  | Yes | 1 |
| Waking up too early | Categorical | No (ref) | 0 |
|  |  | Yes | 1 |
| Daytime naps | Categorical | Yes usually | 2 |
|  |  | Yes, but only in summer | 1 |
|  |  | No (ref) | 0 |

**Table. S3** **Association between chronological age, biological age and all-cause mortality**

|  | HR (95%CI) |
| --- | --- |
| Model 1 |  |
| CA | 1.059 (1.056, 1.062) |
| BA | 1.069 (1.066, 1.073) |
| Model 2 |  |
| CA | 1.058 (1.055, 1.061) |
| BA | 1.068 (1.065, 1.072) |
| Model 3 |  |
| CA | 1.044 (1.040, 1.047) |
| BA | 1.052 (1.048, 1.056) |

NOTE: HR: hazard ratio, CI: confidence interval, CA: chronological age, BA: biological age. Model 1 was built based on CA or BA, model 2 was adjusted for gender, model 3 was adjusted for gender, education attainment, marital status, household income, BMI, smoking, drinking, physical activity, and sleep.

**Table. S4 Association between gender and biological age acceleration**

| Gender | HR (95% CI) | P value |
| --- | --- | --- |
| Male | 1.032 (1.022, 1.043) | < 0.001 |
| Female | 1.030 (1.016, 1.044) | < 0.001 |

NOTE: Multiple linear regression models were employed to examine the relationship between gender and biological age acceleration, with adjustments made for age, educational attainment, marital status, household income, body mass index (BMI), smoking, drinking, physical activity, and sleep.
